# RNA therapy

**DOI:** 10.1038/s12276-023-01051-8

**Published:** 2023-07-10

**Authors:** Young-Kook Kim

**Affiliations:** Department of Biochemistry, Chonnam National University Medical School, Hwasun, Jeollanam-do 58128 Republic of Korea

**Keywords:** Drug discovery, Drug development

RNA therapy is defined as the prevention or treatment of disease using RNA-based molecules. In a previous review, I categorized RNA therapy based on the chemistry of various RNA molecules into the categories of antisense RNA, small-interfering RNA (siRNA), RNA aptamers, and messenger RNA (mRNA)^1^. Each exerts its function by targeting different types of genetic material within cells.

A key advantage of RNA therapy is its ability to target a wide variety of substances in cells. As exemplified by recent drugs targeting cancer, small molecule- and antibody-based drugs are commonly used to treat diseases, and they mainly target proteins by recognizing the specific structure of a target protein^2^. RNA-based drugs, such as those that utilize antisense RNAs and siRNAs, inhibit protein production by targeting mRNA, an intermediate product of protein expression^1^. In addition, RNA aptamers can regulate proteins by binding to them directly. However, the products from non-coding RNA genes, which are more abundant than protein-coding genes in cells^3^, are similar to one another, except for in their sequences; thus, it is difficult to use conventional small-molecule- or antibody-based drugs to target them based on structural differences. In contrast, RNA-based drugs, including antisense RNAs and siRNAs, can bind to target RNAs in a sequence-specific manner, which means that these RNA drugs can suppress non-coding RNAs with similar structures but different sequences.

Diverse non-coding RNAs play important roles in disease progression, and controlling them is important for treating human disease. Regarding non-coding RNAs, the importance of small RNAs in particular has been emphasized in recent years. Specifically, small non-coding RNAs that regulate other molecules are important modulators of various cellular signaling pathways. One of the most intensely researched RNAs is microRNA (miRNA). MiRNAs are single-stranded, small regulatory RNAs of approximately 23 nucleotides that modulate mRNAs, mainly by targeting their 3'-untranslated regions^4^. These miRNAs act as important regulators of human disease progression; cancer was the first disease in which their importance was studied. In an early study to comprehensively profile normal and cancerous tissues using microarrays, the expression patterns of miRNAs were found to clearly distinguish cancerous tissues from normal tissues^5^. Moreover, another study showed that the origin of cancerous tissue can be accurately identified by measuring the expression of miRNAs^6^. Building on these preliminary studies and other important research, various miRNAs that control the oncogenes or tumor suppressor genes important in the progression of diverse cancer types have been identified.

Since then, much research has been performed, and efforts continue to develop therapies that target key miRNAs. To inhibit disease-associated miRNAs, antisense RNA, which can bind directly to miRNAs, can be utilized. This binding prevents miRNA from regulating its target mRNAs. However, if increased expression of a miRNA is expected to inhibit the progression of a particular disease, the miRNA sequence can be synthesized and administered to patients. In this special issue of *Experimental & Molecular Medicine*, Kim & Croce summarize recent and ongoing miRNA clinical trials focusing on cancer diagnosis and therapy^7^.

SiRNAs are another important family of small regulatory RNAs that are mechanistically similar to and share core functional molecules with miRNAs. Although they are expressed endogenously in cells, their roles as regulators have been studied less often than those of miRNAs^8^. Instead, a great amount of work has been conducted with the goal of silencing specific RNAs by introducing chemically synthesized siRNAs into cells. This technique, known as RNA interference, can be used to transiently induce the cleavage of target RNAs that are important in disease progression^9^. Based on this mechanism, many siRNA-based drugs have been developed, and some have already been approved to treat specific diseases^1^.

However, to date, the siRNAs that have been approved or are being studied mainly target enzymes in liver cells. This is because when siRNA is injected into the body, a significant proportion of the molecules are primarily targeted to the liver via circulation^10^. SiRNAs are being actively investigated as therapeutic agents for diverse metabolic diseases related to the liver, especially with the help of hepatocyte-targeting conjugates, such as N-acetylgalactosamine^11^. However, the siRNA targeting of the transcripts residing in other organs has been less well researched because it is challenging to deliver siRNAs to other organs. Therefore, developing siRNA-based drugs that can target various organs is the most important issue in this field, and is a challenge that Han and colleagues discuss in this issue of *Experimental & Molecular Medicine*^12^.

There are many other types of small regulatory RNAs in cells. In particular, transfer RNAs (tRNAs), which were initially known only for their auxiliary roles in transferring amino acids during mRNA translation, have recently been recognized to play important roles as intracellular regulators. The cleavage products of tRNAs, called tRNA-derived small RNAs (tsRNAs), play important roles in the progression of cancer, and this topic is discussed by Kim and colleagues in this special issue^13^.

While RNA-based drugs to target these various small regulatory RNAs are in development, it is perhaps the mRNA vaccine for coronavirus disease 2019 (COVID-19) that has spurred major research interest in RNA therapy. It was possible to develop an mRNA-based vaccine to counter the COVID-19 pandemic because mRNA-based materials can be developed quickly. Moreover, once the chemistry of the material has been established and the synthesis platform is in place, RNA-based drugs can be easily adapted to the treatment of other types of diseases by changing their RNA sequences^1^. Based on these strengths, various mRNA-based vaccines against a wide range of infectious diseases are currently being developed^14^. However, as seen in the case of the COVID-19 vaccine, many people who have received the vaccine have complained of side effects, which is a phenomenon requiring further study. In this special issue of *Experimental & Molecular Medicine*, Lee et al. describe immune responses and adverse effects triggered by mRNA vaccines and discuss the challenges in balancing these effects^15^.

As shown by recent studies, the field of RNA therapy has made significant progress. In particular, the ability to target various genetic materials in the body and to develop drugs quickly are reasons to believe that more research efforts will be focused on the development of RNA-based therapeutics in the future. It is expected that by developing better drugs, it will become possible to overcome diseases that were previously difficult to treat and prevent.
